# Quantitative analysis of multi-components by single marker method combined with UPLC-PAD fingerprint analysis based on saikosaponin for discrimination of Bupleuri Radix according to geographical origin

**DOI:** 10.3389/fchem.2023.1309965

**Published:** 2024-01-19

**Authors:** Yuting Li, Xiaoli Wu, Yuzhi Ma, Lijia Xu, Chengmin Yang, Dongqin Peng, Xinwei Guo, Jianhe Wei

**Affiliations:** ^1^ Key Laboratory of Bioactive Substances and Resources Utilization of Chinese Herbal Medicine, Ministry of Education and National Engineering Laboratory for Breeding of Endangered Medicinal Materials, Institute of Medicinal Plant Development, Chinese Academy of Medical Sciences and Peking Union Medical College, Beijing, China; ^2^ State Key Laboratory of Aridland Crop Science, Gansu Agricultural University, Lanzhou, Gansu, China

**Keywords:** Bupleuri Radix, quantitative analysis of multi-constituent by a single marker (QAMS), fingerprint, saikosaponin, *Bupleurum chinense* DC

## Abstract

**Background:** Saikosaponins are regarded as one of the most likely antipyretic constituents of Bupleuri Radix, establishing a comprehensive method that can reflect both the proportion of all constituents and the content of each saikosaponin is critical for its quality evaluation.

**Methods:** In this study, the combination method of quantitative analysis of multiple components with a single marker (QAMS) and fingerprint was firstly established for simultaneous determination of 7 kinds of saikosaponins in Bupleuri Radix by ultra-high performance liquid chromatography (UPLC).

**Results:** The results showed that saikosaponin d was identified as the optimum IR by evaluating the fluctuations and stability of the relative calibration factors (RCFs) under four different conditions. The new QAMS method has been confirmed to accurately quantify the 7 kinds of saikosaponins by comparing the obtained results with those obtained from external standard method and successfully classify the 20 batches of Bupleuri Radix from 8 provinces of China. The experimental time of fingerprint was significantly reduced to approximate 0.5 h through UPLC-PAD method, a total of 17 common peaks were identified.

**Conclusion:** The QAMS-fingerprint method is feasible and reliable for the quality evaluation of Bupleuri Radix. This method could be considered to be spread in the production enterprises of Bupleuri Radix.

## 1 Introduction

Bupleuri Radix is one of the most frequently used traditional Chinese medicines (TCMs). Since 1600s AD (the Qing Dynasty in China), the most widely planted and consumed species is *Bupleurum chinense* DC (Wang et al., 2021). As the main species in Xiao-Chai-Hu decoction and the effective herbal formula Qingfei Paidu decoction (QFPD) used for the treatment of COVID-19 (Chen et al., 2020), *B. chinense* DC. has been used as an antipyretic medicinal herb for more than 2000 years. However, so far, the material basis for its antipyretic effect is still unclear; and thus, the extract of Bupleuri Radix has always been used in clinical application, rather than any of its monomer compounds (Kang et al., 2023). Therefore, similar as many other TCMs, the quality control of Bupleuri Radix is with the feature of integrity and diversity, and one or two indicators cannot represent the whole quality of the medicines (Xu et al., 2017).

Phytochemical studies showed that *B. chinense* DC. contains triterpene saponins, volatile oils, saponins, polyacetylenes, flavonoids, lignans, fatty acids, and sterols (Sun et al., 2018; Yang et al., 2020; Li et al., 2022; Teng et al., 2023), of which triterpene saponins were account for 7% of the total dry root weight and were regarded as one of the most likely antipyretic constituents (Pistelli et al., 1993; Ebata et al., 1996; Sui et al., 2020). In the Chinese Pharmacopoeia, saikosaponin a and saikosaponin d are recognized as the quality control indicators of Bupleuri Radix (State Pharmacopoeia Commission, 2020). Besides, saikosaponin b has been reported to involve in regulating the body’s immune response (Zhang et al., 2017); saikosaponion b_2_ and saikosaponion c have been known to play roles in anti-viral response (Chiang et al., 2003; Lin et al., 2015); although the pharmacological effects of other saikosaponions such as saikosaponion e and saikosaponion f are not clear, their content in Bupleuri Radix is also not low (Yoko et al., 2011). As it is not yet confirmed that saikosaponin is the key constituent that responsible for antipyretic effect, establishing a quality evaluation method that can simultaneously reflect the proportion of all constituents and the content of each saikosaponin is critical for Bupleuri Radix.

Currently, the combination of Quantitative analysis of multi-constituent by a single marker (QAMS) and fingerprint method has become one of the most commonly used strategy for the quantitative and qualitative evaluation of TCMs (Zou et al., 2008; Cao et al., 2022), QAMS method requires selecting an easily available active constituent as internal reference standard (IRS), which can be used to calculate the contents of multiple constituents (Wang et al., 2006), and thus solves the bottleneck problem of the scarcity of reference substances and reduce the high cost and time of detection. On the other hand, chromatographic fingerprints technique is the most important method for evaluating the quality of multi-constituent TCM by comprehensively determining almost all detected common peaks (Wang et al., 2020). The combination of QAMS and chromatographic fingerprints technique enable simultaneous determinations of multiple target constituents and common peaks, which could largely improve the efficiency and comprehensiveness of the quality control of TCMs. Nowadays, the QAMS-fingerprint method has been successfully established and applied in many TCMs, such as Psoraleae Fructus (Luan et al., 2018), Radix Astragali (Huang et al., 2018), Rhubarb (Dou et al., 2021), Berberidis Cortex (Su et al., 2022), and Pulsatilla Chinensis (Chen et al., 2022), but there has been no relevant report in Bupleuri Radix. In addition, the current fingerprint method for Bupleuri Radix has a detection time of up to about 1.5 h, which is unable to applicate (Zhou et al., 2022). Thus, establishing a combination method of QAMS-fingerprint is of high importance in the quality evaluation of Bupleuri Radix.

In terms of the methodology for analysis in Bupleuri Radix, the reported methods mainly include HPLC-UV, HPLC-ELSD, HPLC-CAD, HPLC-DAD and UPLC. Due to the weak absorption of UV, the analysis of saikosaponins by HPLC-UV has the limitations of low sensitivity and unstable although acidification treatment of the sample has been proven to have an optimization effect, the above problems still cannot be greatly improved; ELSD is a general detector, but its sensitivity and repeatability are not high enough. HPLC-CAD and HPLC-DAD could significantly improve the above problems, but the cost of these 2 instruments is too high to be applied. Compared with HPLC, UPLC has the advantages of high sensitivity and good separation efficiency. It could largely shorten analysis time and reduce analysis costs, and have been proved to be suitable for the analysis of saikosaponins of Bupleuri Radix.

In this study, we aim to establish a simple, economical, and efficient QAMS-fingerprint method that focus on 7 kinds of saikosaponins in Bupleuri Radix. 20 batches of the samples were collected from different production regions of China. The extraction procedure and UPLC method were optimized. The newly established QAMS method was validated and evaluated by comparing the obtained results with those obtained from conventional external standard method (ESM) (Yi et al., 2018). Based on PCA and HCA analysis, this QAMS method was also tested to determine whether it can be used as fingerprint to successfully discriminate the 20 samples. The optimum IR was used to calculate the average relative calibration factors (RCFs). The fluctuations and stability of the RCFs were evaluated under different conditions. Overall, in response to the current situation of unclear quality indicators, our newly established QAMS-fingerprint method provides a reliable, comprehensive and efficient way for the quality evaluation of Bupleuri Radix.

## 2 Materials and methods

### 2.1 Chemicals and materials

**FIGURE 1:**
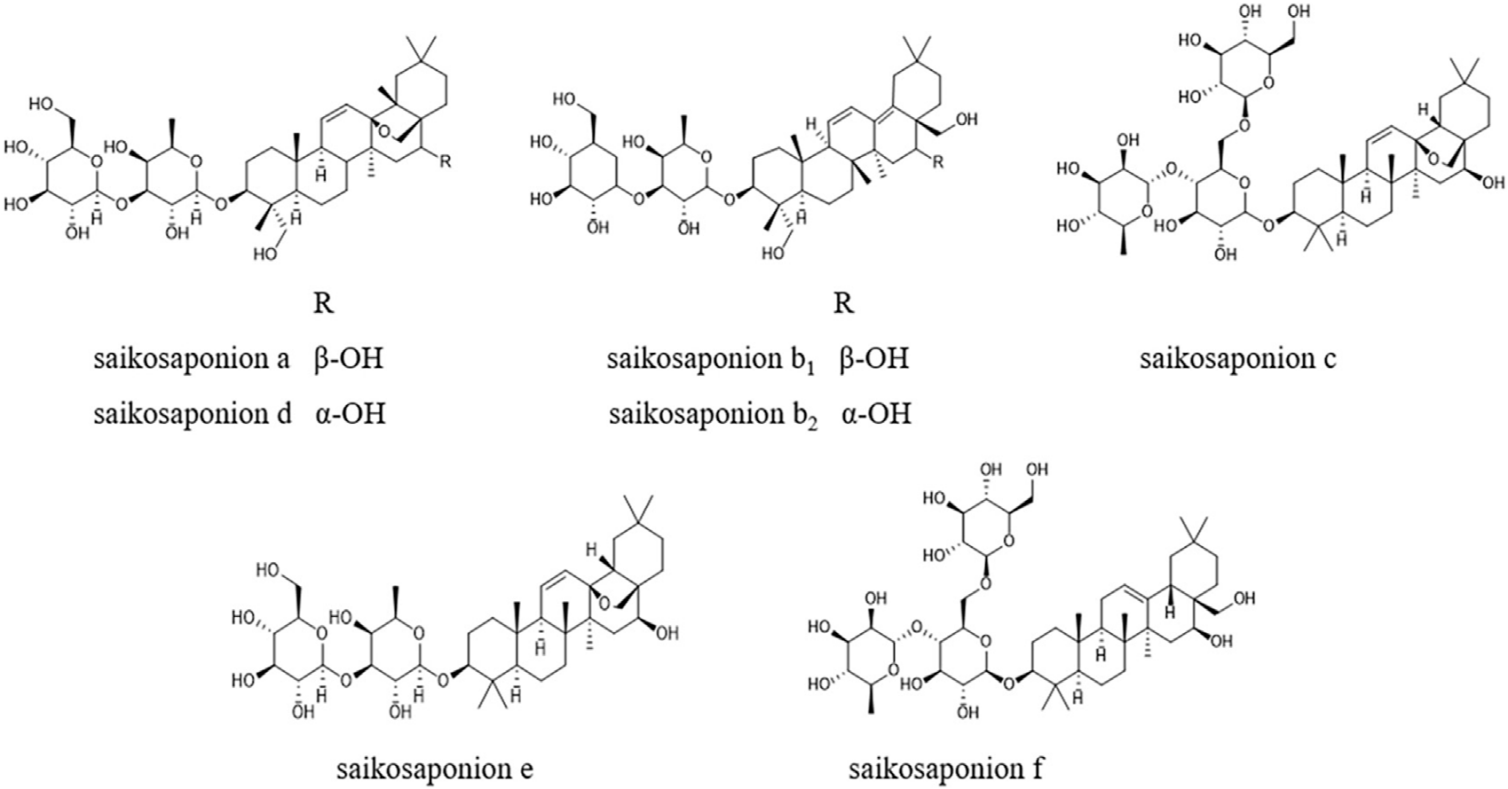
The chemical structures of seven standards.

The standards of saikosaponion a (110777, purity ≥91.1%) and saikosaponion d (110778, purity ≥95.8%) were originally purchased by the company of National Institutes for Food and Drug Control; the standards of saikosaponion b_1_ (B20147, purity ≥98.0%) and saikosaponion c (B20147, purity ≥98.0%) were originally purchased by Yuanye Bio-technology Company, Ltd. (Shanghai, China); the standards of saikosaponion b_2_ (S861351, purity ≥98.0%), saikosaponion e (S914367, purity ≥98.0%) and saikosaponion f (S873544, purity ≥98.0%) were originally purchased by Macklin Biochemical Company, Ltd. (Shanghai, China). The basic chemical structures and compositions of these compounds are shown in Figure 1. Acetonitrile and methanol were purchased from Thermo Fisher Scientific Company, Ltd. (Shanghai, China) (HPLC grade). Ammonium hydroxide was from Beilian Fine Chemicals Development Company, Ltd. (Tianjin, China) (Analytical grade). Purified water was purchased from Watsons Food & Beverage Company, LTD. (Hongkong, China). In addition to these, all other chemicals used in this study were of analytical grade.

In this study, the collected 20 batches samples were from different production areas as shown in Table 1. The chosen planting areas represent most of the major regions in China where these plants are planted. The samples were authenticated by DNA barcoding identification technology, and the bases are respectively *B.chinense* DC (Supplementary Figure S1).

**TABLE 1:** The production areas of *B. chinense* DC.

No.	Production areas	Longitude/¡ã	Latitude/¡ã	Sample source
S1	Baoji, Shaanxi	107.735	34.356	Planting Base
S2	Baoji, Shaanxi	107.735	34.356	Market
S3	Chengde, Hebei	117.340	40.948	Planting Base
S4	Luoyang, Henan	112.092	34.141	Planting Base
S5	Jiangxian, Shanxi	111.575	35.497	Planting Base
S6	Jishan, Shanxi	110.989	35.610	Planting Base
S7	Shiyan, Hubei	111.734	32.047	Planting Base
S8	Haidian, Beijing	116.305	39.965	Planting Base
S9	Baokang, Hubei	111.234	31.749	Market
S10	Qingyang, Gansu	107.649	35.715	Market
S11	Wanrong, Shanxi	110.845	35.421	Market
S12	Wenxi, Shanxi	111.231	35.363	Market
S13	Chifeng, Inner Mongolia	118.896	42.262	Market
S14	Liangcheng, Inner Mongolia	112.511	40.537	Market
S15	Chengcheng, Shaanxi	109.938	35.197	Market
S16	Qingyang, Gansu	107.649	35.715	Planting Base
S17	Dingxi, Gansu	104.632	35.587	Planting Base
S18	Chifeng, Inner Mongolia	118.896	42.262	Planting Base
S19	Pingshun, Shanxi	113.443	36.206	Planting Base
S20	Fangshan, Beijing	116.149	39.754	Planting Base

### 2.2 Designing of the experiment

In this study, we established an accessible QAMS combined with fingerprinting method by UPLC for the quality evaluation of *B.chinense* DC. Firstly, the structures of the 7 target compounds were characterized and identified by UHPLC-Q-TOF-MS and the existing extraction conditions were optimized and the extraction rates under different conditions were examined separately to determine the optimal extraction conditions. Second, the validation of the method should be verified by a series of experiments, such as linearity, reproducibility, stability, precision, limit of detection (LOD), limit of quantification (LOQ) and spiked recovery of the method. Third, different injection concentrations were used to calculate the RCF values, and the stability of RCF under different conditions (column, column temperature, mobile phase flow rate, and injection volume) was investigated. Fourth, based on the relative deviations (RE) between the QAMS and the ESM, the relative adaptability and feasibility of this new methodology was assessed.

### 2.3 Conditions of chromatographic and mass spectrometric

The analysis of the experiment was conducted by using the equipment of Waters Acquity UPLC H-Class liquid chromatographic system, which consists of an auto-sampler manager, a photo diode array detector, a quaternary solvent delivery pump, and connects the Waters Empower3 software. The Waters ACQUITY UPLC BEH C_18_ columns (100 mm × 2.1 mm, 1.7 μm) was used to separate the samples. The acetonitrile (A) and water (B) was composed of the mobile phase with a gradient elution of 25%–35% A (0–5 min), 35%–38% A (5–8 min), 38% A (8–14 min), 38%–60% A (14–18 min), 60%–82% A (18–28 min), 82%–90% A (28–31 min). The injection volume was 2 μL. The flow rate was set at 0.3 mL/min, the column temperature was set at 35°C. And 210 nm and 254 nm was set as the detection wavelength simultaneously.

The detection and confirmation of chromatographic peaks were carried out by SYNAPT G1 mass system with electrospray ionisation and MassLynx (V4.1) software. The mass spectra were acquired in negative mode by scanning from 100 to 1500 in mass to charge ratio (m/z). The MS analysis was performed under the following operation parameters: dry gas temperature 450°C, dry gas (N2) flow rate 900 L/h, capillary voltage of 3 kV, cone voltage of 30 V.

### 2.4 Sample solutions preparations

The powder of the samples was obtained by crushing the roots of the Bupleuri Radix and sifting from a 65-mesh drug sieve, accurately weigh approximately 1.0 g of the ground sample powder, and put it into a 50 mL stoppered conical flask with 25 mL 5% ammonia methanol mixture, then ultrasonication for 30 min using ultrasonication (KQ-500DE CNC Ultrasonic Cleaner, Kunshan Ultrasonic Instruments Co., Ltd.) at 30°C, 400W and 40 kHz to perform the samples extractions. Waiting for the temperature drop to the room temperature, making up the loss weight with ethanol, and filtered. Each sample should be filtered by 0.22 μm filter membrane, and stored at −20°C before analysis.

### 2.5 Preparation of standard solutions

Accurately weigh the appropriate amount of reference substance of saikosaponin a, saikosaponion b_1_, saikosaponinb_2_, saikosaponion c, saikosaponin d, saikosaponin e and saikosaponin f, and dissolved them into methanol accordingly, then diluted the obtained mixture of standards to the appropriate concentration ranges. The calibration curves were plotted with the concentration as the *X*-axis and the peak area as the *Y*-axis. All solutions were stored at −20°C.

### 2.6 The development of the QAMS method

In this study, minor modifications have been performed according to the available methodology to establish the QAMS method. First of all, it is necessary to select the suitable IR, each standard was used as an IR to calculate the RCFs of other standards in different conditions, and used it to verify the stability of RCFs. Then, an inexpensive and readily available standard that can provide the lowest RSD values under different conditions was used as the internal standard.

### 2.7 Quantitative analysis of multi-components by single marker

The quality control of Bupleuri Radix was performed by the QAMS method, which was according to the proportional value of each standard to the detector within a certain range to calculate RCFs. RCFs of the analyte were determined by using 7 kinds of standards as IRs for different chromatographic columns (Waters ACQUY UPLC BEH C_18_ (100 mm × 2.1 mm, 1.7 μm) columns and Waters ACQUITY UPLC HSS T_3_ (100 mm × 2.1 mm, 1.7 μm) columns), flow rates (0.3, 0.32, 0.34 and 0.36 mL/min), column temperature (28, 32, 35°C and 38°C), sample volumes (1, 2, 3 and 4 μL). The final determination of IRs and RCFs needs to be considered from 2 aspects: first, the selected IRs should be economical, stable, easy to obtain, have certain pharmacological activity, and have good separation degree under appropriate chromatographic conditions; second, the selected IRs should obtain the minimum RSD value.

The RCF was calculated as follows

$$F_{si}=\frac{F_{s}}{F_{i}}=\frac{C_{s}/A_{s}}{C_{i}/A_{i}}=\frac{A_{i}\times C_{s}}{A_{s}\times C_{i}}$$ (1)

*A*_*s*_ (the chromatographic peak areas of each IR), *A*_*i*_ (the chromatographic peak areas of each analyte); *C*_*s*_ (the measured concentrations of each IR (mg/mL)), *C*_*i*_ (the measured concentrations of each analyte (mg/mL)). According to Eq. 1, the measured concentration of component i (*C*_*i*_) can be calculated by Eq. 2.

$$C_{i}=\frac{A_{i}\times C_{s}}{A_{s}\times F_{si}}$$ (2)

### 2.8 Quantitation of seven main saikosaponins in *B. chinense* DC

*W*_*i*_ means the content of saikosaponin i, it can be calculated according to Eq. 3.

$$W_{i}=\frac{C_{i}\times V}{M_{m}}$$ (3)

*V* [the extract volume (mL)], *M*_*m*_ [the extract mass (g)].

### 2.9 Scientific analysis of the data

All samples were measured in triplicate. Excel 2019 was used for statistical analysis of the obtained data, and the final content value of each sample was based on the average value of the 3 measurements. With the content of 7 kinds of compounds as variables, cluster analysis was performed on 20 batches of the samples. And SPSS (27.0.1) and SIMAC (14.0) were used for HCA and PCA analysis, respectively.

Fingerprinting of Chinese herbal medicines refers to the chromatogram or spectrogram that can indicate the chemical characteristics of certain Chinese herbal medicines or Chinese medicinal preparations after appropriate treatment and the use of certain analytical means. In this study, 20 batches of samples were analyzed for similarity using the TCMs chromatographic fingerprinting software.

## 3 Results and discussions

### 3.1 Optimization of sample pre-treatment

According to previous research by scholars, sample pretreatment has a certain impact on the experimental results (Yudthavorasit et al., 2014) Therefore, the way in how the sample is pre-treated is very important for the analysis of the sample. In this study, the extraction method was optimized under Chinese Pharmacopoeia for genus *Bupleuri.* The effects of extraction time (20, 25, 30 and 35 min), extraction power (200w, 300w, 400w and 500 w), extraction solvent (5% ammonia-methanol, methanol, 10% ammonia-methanol) and extraction temperature (20°C, 25°C, 30°C and 35°C) on the extraction rate were investigated respectively. The best extraction conditions were finally determined to be 5% ammonia methanol, 35°C, 400w and 30 min (Supplementary Table S1), under which the extraction rate was higher and more reproducible.

### 3.2 Establishment of UPLC method

#### 3.2.1 UPLC conditions

**FIGURE 2:**
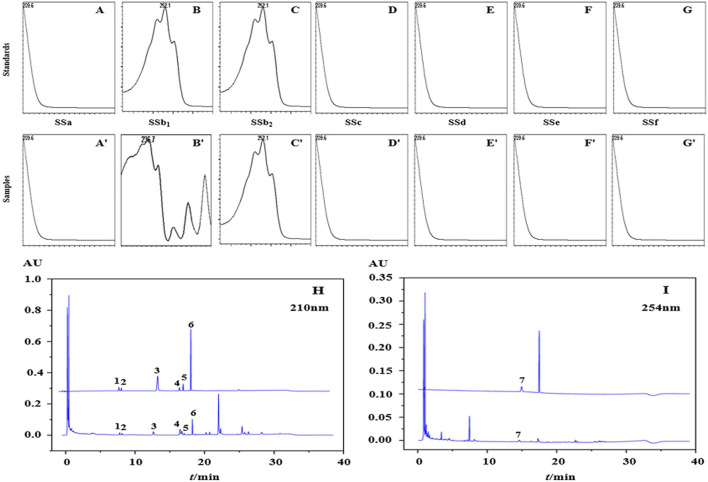
The PDA spectrum of each analytes in the mixture of standards **(A–G)** and samples **(A**′**–G**′**)**. The chromatograms of the mixture of standards and samples in the wavelength of 210 nm **(H)** and 254 nm **(I)**.

In this study, 7 kinds of target compounds were not detected at the same time as other substances, indicating that the experimental error was small and acceptable for subsequent experiments. The wavelength of 254 nm was chosen for the quantification of saikosaponion b_2_ and 210 nm for the quantification of the remaining 6 substances. Under this gradient procedure, a good separation of almost all substances was achieved within 31 min with a stable baseline and no peak interference. The chromatograms are shown in Figure 2 and peaks 1-7 were identified based on the retention times of the reference chromatograms as saikosaponion c, saikosaponion f, saikosaponion a, saikosaponion b_1_, saikosaponion e, saikosaponion d and saikosaponion b_2_. And the UHPLC-Q-TOF-MS also showed the same results (Supplementary Figures S2–S9), 7 target compounds were identified as saikosaponion c, saikosaponion f, saikosaponion a, saikosaponion b_1_, saikosaponion e, saikosaponion d and saikosaponion b_2_ separately, which were the same as the previous study (Zhu et al., 2017).

#### 3.2.2 Validation of the method specificity

The specificity of the detection method is evaluated by detecting the purity of the peak. This is conducted by comparing the retention time of the target compound with the consistency of the PDA spectrum between the sample and the mixture of standards. Figure 2 shows the peak purity of each analytes using a photodiode array detector (PDA).

### 3.3 Method validation

#### 3.3.1 Calibration curves linearity

The calibration curves were plotted for 7 kinds of standards using a range of standard solutions with a concentration gradient. Table 2 shows the calibration curves of 7 kinds of compounds in a certain range, and their correlations are all greater than 0.9990 (*R* ^2^ > 0.9990), indicating that each analytes has a good linear relationship, and the Table 2 also shows the LOD and LOQ of each target compound. LOD and LOQ are the absolute quantities of the measured substance when the signal-to-noise ratio is 3 or 10, respectively.

**TABLE 2:** The Regression Equation, Linear Range, and Stability of the 7 kinds of Components Analyzed by UPLC.

Reference substances	Rt	Regression equation	R^2^	Liner range	LOD	LOQ
	min			μg/ml	μg/ml	μg/ml
saikosaponion a	13.838	y = 2015.2x-10091	0.9995	7.60-400	0.8	2.7
saikosaponion b1	17.176	y = 2679.1x-2189	0.9999	3.10-100	0.3	0.9
saikosaponion b2	14.407	y =9033.5x-823.95	0.9995	0.60-14.4	0.01	0.05
saikosaponion c	8.553	y = 1255.6x-1936.3	0.9996	16.0-243	0.2	0.6
saikosaponion d	18.937	y= 2132.2x-5982.5	0.9997	31.7-475	0.9	3.0
saikosaponion e	17.803	y= 1994.3x-991.36	0.9999	4.00-83.0	0.17	0.6
saikosaponion f	8.871	y = 1357.7x-865.23	0.9991	4.80-150	0.22	0.7

#### 3.3.2 Precision

A mixed control solution of a certain concentration was aspirated precisely and sampled 7 times on the same day according to the chromatographic conditions. The peak areas of the 7 kinds of target compounds were recorded and the RSD values were calculated to evaluate the intra-day precision; the peak areas of each component were recorded and the RSD values were calculated as the inter-day precision for 3 consecutive 3-d, daily injection analysis. The results are shown in Table 3. The intra-day and inter-day precision RSD values were less than 3%.

**TABLE 3:** Accuracy, Precision and recovery of the 7 kinds of Components.

Compounds	Intra-Day	Inter-Day	Stability	Repeatability	Recovery (*n* = 3)					
	RSD/%, *n* = 6	RSD/%, *n* = 9	RSD/%, *n* = 6	RSD/%, *n* = 6	Added level	Recovery (%)	Added level	Recovery (%)	Added level	Recovery (%)
saikosaponin a	1.6	1.1	2.1	2.4	Low	102.6	Middle	102.7	High	104.0
saikosaponin b1	1.7	0.8	2.7	2.4	Low	99.3	Middle	98.8	High	101.1
saikosaponin b2	1.8	1.4	2.4	2.7	Low	98.5	Middle	103.2	High	102.4
saikosaponin c	1.5	1.2	1.8	0.6	Low	98.5	Middle	102.3	High	99.7
saikosaponin d	1.5	0.8	1.7	2.5	Low	98.9	Middle	102.5	High	100.7
saikosaponin e	1.1	0.9	1.4	1.4	Low	99.1	Middle	100.9	High	98.5
saikosaponin f	1.7	1.7	1.5	2.1	Low	100.1	Middle	97.3	High	100.4

#### 3.3.3 Stability

We investigated whether the sample solutions were stable when stored at room temperature for 24 h. The stability of the sample solution (S1) at room temperature was tested at 0, 2, 4, 6, 8, 12 and 24 h to obtain the RSD. The RSD values for the stability experiments are shown in Table 3 and the RSD% was less than 3%. The method can be considered stable and the sample solution was stable over 24 h.

#### 3.3.4 Repeatability

Six test solutions were prepared in parallel and analyzed by chromatography under the following conditions, and the contents and RSD values of the 7 kinds of components to be tested were calculated. The results showed in Table 3 and the RSD values were below 3%, indicating that the method was reproducible.

#### 3.3.5 Accuracy

To verify the accuracy of the study, a mixed standard solution of 80%, 100%, 120% concentrations of the analyte was added to a certain amount (1.0 g) of sample (S1) and repeated 3 times. The mixture was then extracted and analyzed, the mean spiked recoveries of the seven components were calculated and the RSDs of the precision values are shown in Table 3.

#### 3.3.6 Establishment of internal reference material (IR)

The key to the establishment of the QAMS method is the selection of a suitable IR and the verification of the stability of the RCFs calculated from this IR under different conditions.

**FIGURE 3:**
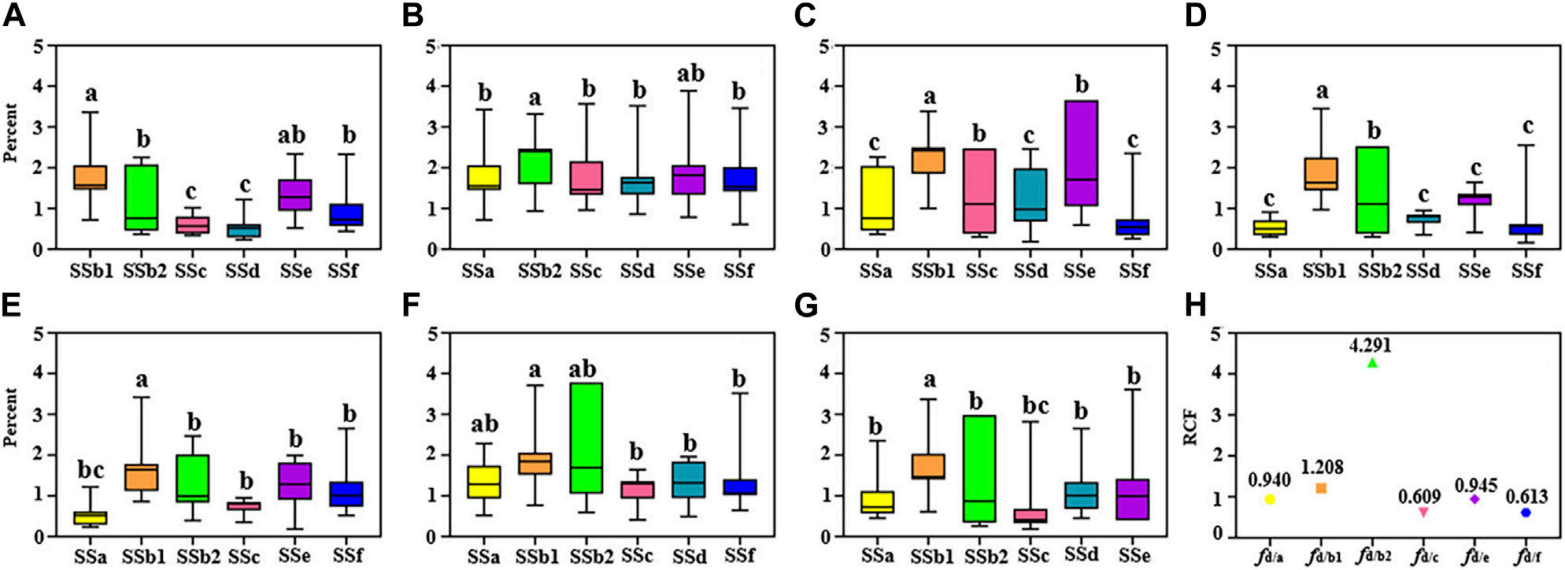
Boxplots of RCF under different conditions by using different internal references (IRs): the RSDs of seven target components with saikosaponin a, saikosaponin b_1_, saikosaponin b_2_, saikosaponin c, saikosaponin d, saikosaponin e, saikosaponin f as IR **(A–G)**; the RCF in determined conditions with saikosaponin d as an IR **(H)**.

In previous studies, there were no scholars have researched the selection of internal standards in the Bupleuri Radix QAMS method, and the stability of RCFs under different conditions has not been comprehensively investigated. Therefore, in this study, we examined different conditions (different column temperatures, different flow rates, different columns and different injection volumes) to determine the optimum IR. For a more visual presentation of the data, RSD box plots were used (Figure 3). The RCFs of the analyte is calculated according to the Eq. 1 under different study conditions. In order to select the suitable IRs, the principle of selection is according to the RSD values (usually RSD <5% indicates that the error has a small effect on the RCF). Figures 3A–G show that the RSD of the RCFs for all standards were below at 5%, indicating that the RCFs were reproducible and 7 target standards can be regarded as quantitative IRs. In summary, the above results indicate that the errors caused by different investigation conditions are small and provide a basis for further testing of the stability of the RCFs. In addition, saikosaponin d was found to be more abundant and readily available in Bupleuri Radix and met the selection criteria for IR. Therefore, saikosaponin d was selected as the IR to quantify the other five compounds. For these five components, the RCF deviation was less than 3%, indicating that the QAMS method can be used for quantitative analysis with saikosaponin d as IR.

#### 3.3.7 Calculation of the relative accuracy factor

**FIGURE 4:**
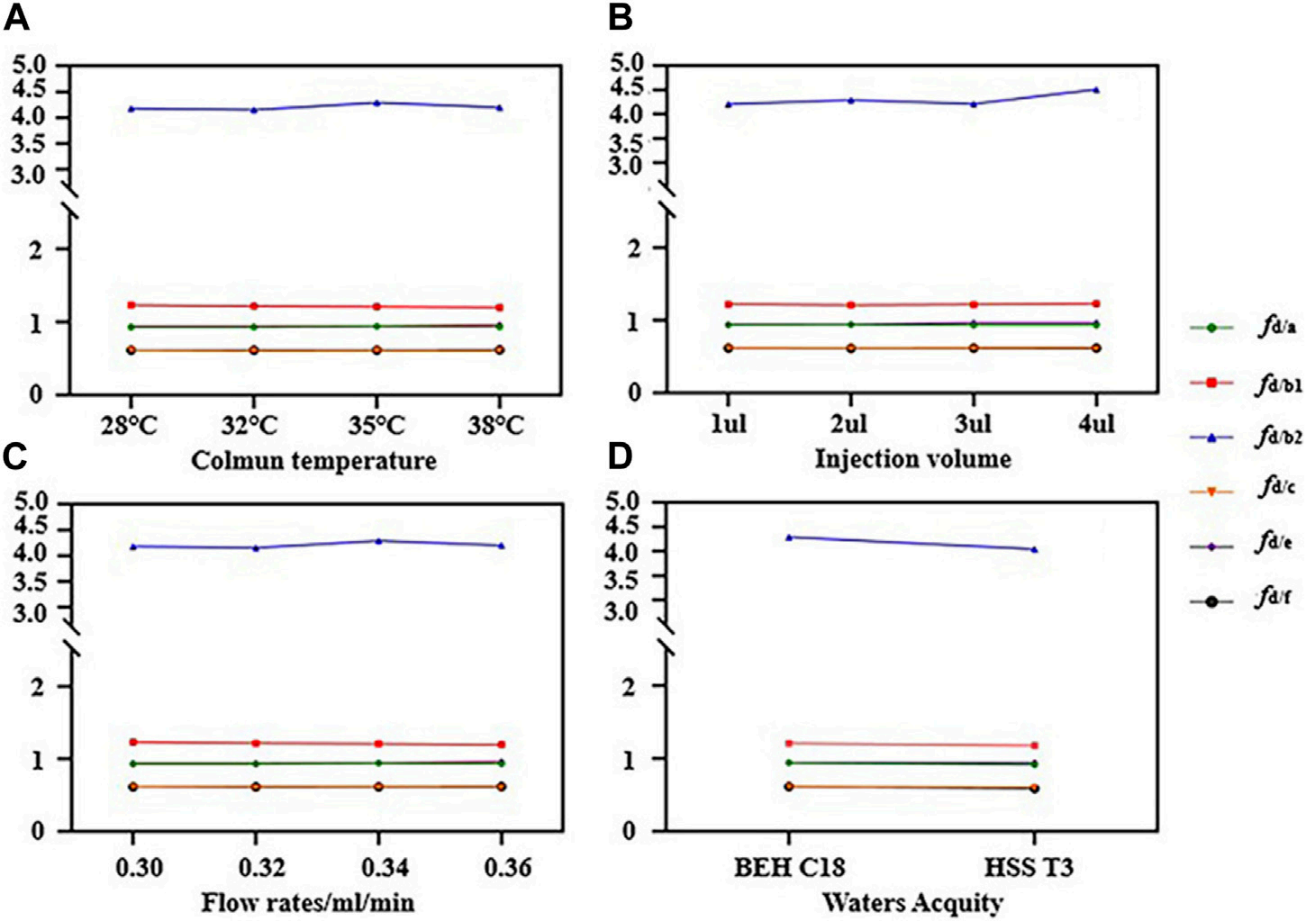
Results of relative calibration factors (RCFs) in different conditions **(A–D)**. **(A)** RCFs of different column temperatures; **(B)** RCFs of different injection volumes; **(C)** RCFs of different flow rates; **(D)** RCFs of different columns.

The RCFs of saikosaponin d as IR under different conditions were determined by examining the RCFs of different standards as IRs under different conditions, Figure 4 presents the RCFs of saikosaponin d as IR under different conditions in the form of a line graph. The RCFs at each gradient were calculated from a mixture of standard substances in a series of concentration gradients (Eq. 1), and the average RCFs was then used as the final RCF for each compound. It can be seen that the IR does not change significantly under different conditions, indicating that the QAMS method has the advantage of being highly stable.

### 3.4 Quantitative analysis by QAMS and ESM

As known, the extraction and purification process of some standard samples of saikosaponins is complex and difficult to obtain. In this study, due to the relatively cheap price of saikosaponin d, which is only about 1/4 of saikosaponin e, and the highest content of saikosaponin d in Bupleuri Radix, which is easy to determine, we chose saikosaponin d as the internal standard to calculate the content of other compounds. The feasibility of the QAMS method was tested by using the traditional ESM method to determine the same batches of samples. The concentration and content calculations under the QAMS method refer to Eqs 2, 3, while those under the ESM method refer to the calibration curve. As shown in Supplementary Table S2, the RE between the QAMS method and the ESM method was lower than 5%, and there was no significant difference in the content of 6 compounds calculated between the two methods, indicating that the established QAMS method could replace the ESM method for quantitative analysis of Bupleuri Radix. The content of the 7 kinds of saikosaponins in the 20 batches of samples showed significant differences, with higher levels in sample S4, S5, S9 and S12, while lower levels in sample S3, S8, and S20.

Therefore, the QAMS method we have established is reliable and could greatly reduce detection costs and improve detection efficiency. Moreover, in this study, we collected almost all the production areas of the *B. chinense* DC., and investigate the RSD of IRs under different conditions to select the suitable IR and verify the stability of the IRs.

### 3.5 Cluster analysis

**FIGURE 5:**
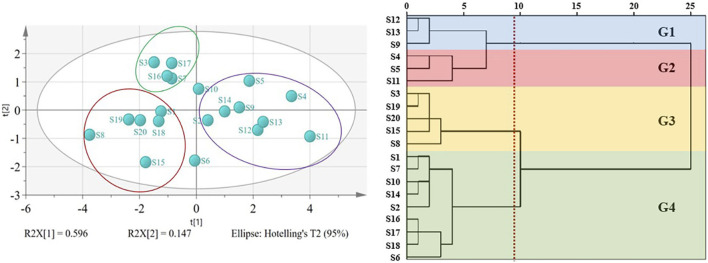
PCA and HCA cluster of 7 kinds of chemical compositions for B chinense DC. of different origins. G1: group 1; G2: group 2; G3: group 3; G4: group.

On the basis of the accurate quantification of the 7 kinds of saikosaponins in the 20 batches of samples, we further performed cluster analysis by using PCA and HCA methods. Overall, the results of the two analysis methods were roughly consistent, namely, the green group, purple group and red group in the PCA results roughly correspond to the G4 group, G1 and G2 groups, and G3 group in HCA analysis, respectively (Figure 5). The G4 group in HCA analysis was the group with the highest degree of sample mixing, including samples S7, S16, and S17 from the green group, samples S2, S10, and S14 from the purple group, and samples S18 from the red group in PCA analysis. However, due to the close distance between these samples under PCA analysis, we believe that this deviation is within an acceptable range.

We further analyzed the above clustering results based on the longitude and latitude of the sampling locations (Table 1). Taking the PCA results as an example, although the samples in each group come from different provinces, the majority of the samples from the green and the purple groups have the latitudes lower than N 35°, while the majority of the samples from the red group have the latitudes higher than N 35°. No significant differences were found in longitude.

It is known that the quality of TCMs is closely related to the environment. Previous studies showed that saikosaponins, ginsenoside and other terpenoids are very sensitive to temperature, that is, to some extent, latitude has more influence on the synthesis and accumulation of terpenoids than longitude (He et al., 2022). As shown in Table 4, the levels of the 7 kinds of saikosaponins of the red group (such as the sample S3, S8 and S20) which from the higher latitude was lower than that of the groups from the lower latitudes (such as the sample S4, S5, S9 and S12). Thus, it is indicated that different from the situation that low temperature promotes the accumulation of ginsenoside (Zhao et al., 2019), the north temperate zone with moderate temperature might be most conductive for the accumulation of saikosaponins, which is also consistent with the view that the Baoji area in southern Shaanxi is the genuine producing area of Bupleurum Radix.

**TABLE 4:** The similarity of 20 batches samples from different production areas.

No.	Similarity	No.	Similarity
S1	0.908	S11	0.855
S2	0.815	S12	0.715
S3	0.950	S13	0.845
S4	0.896	S14	0.944
S5	0.946	S15	0.640
S6	0.882	S16	0.883
S7	0.881	S17	0.767
S8	0.776	S18	0.898
S9	0.796	S19	0.697
S10	0.811	S20	0.822

### 3.6 Fingerprint analysis

**FIGURE 6:**
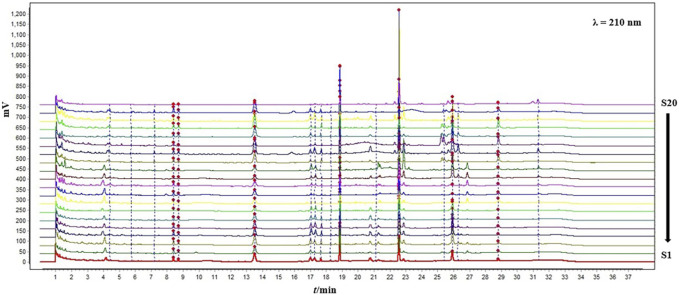
The fingerprint chromatograms of the 20 samples from different production areas at 210 nm.

Fingerprint is an important technique that comprehensively reflects the overall quality of TCMs. Most of the established fingerprint spectra of Bupleurum chinense DC. are HPLC methods, and its experimental time is generally longer than 80 min. This study sharply shortened the entire experimental time to about 30 min, and used the UPLC method, greatly improving the efficiency and increasing the sensitivity of the method. As shown in Figure 6, a total of 17 common peaks were finally identified. The result of similarity analysis was shown in Table 4. Different with the PCA or HCA analysis of saikosaponins, no correlation was found between the results of fingerprint and latitude or longitude. In addition, significant differences existed among the samples from the same province, or even the same region, which may be caused by multiple factors such as germplasm, environment, and processing procedure. While, the samples from the high-quality production areas of Bupleuri Radix, including Chengde (S3), Jiangxian (S5), Baoji (S1), Songxian (S4), Jishan (S6) and Shiyan (S7), exhibited a high degree of similarity of 85% to over 90%, confirming the reliability of this QAMS-fingerprint method.

Besides, the significant difference between the results of fingerprint and PCA/HCA analysis once again indicated that using saikosaponins as the single representative constituent for quality evaluation of Bupleuri Radix is not enough. In this study, since the QAMS method is generally aimed at only one kind of component, we first paid attention to saikosaponins, the most active constituent of Bupleuri Radix. In the future study, we will focus on volatile oil, flavonoid and other important components.

## 4 Conclusion

In summary, for the first time, we established an efficient determination method combining QAMS and fingerprint analysis and successfully determined 7 kinds of saikosaponins in *B. chinense* DC. saikosaponin d was identified as the best IR, and the detection time of the fingerprint analysis was significantly shortened. The relevant results showed that the above method was stable and reliable. This new established method will provide a strong basis for the quality evaluation of *B. chinense* DC., and will also provide important reference for the rapid evaluation of multiple saponins in other related TCMs.

## Data Availability

The original contributions presented in the study are included in the article/Supplementary Material, further inquiries can be directed to the corresponding authors.
