# Supplementary figures and images for: Quantitative analysis of multi-components by single marker method combined with UPLC-PAD fingerprint analysis based on saikosaponin for discrimination of Bupleuri Radix according to geographical origin
Source: Front Chem. 2024 Jan 19;11:1309965. doi: 10.3389/fchem.2023.1309965 (PMC10834642; doi:10.3389/fchem.2023.1309965)

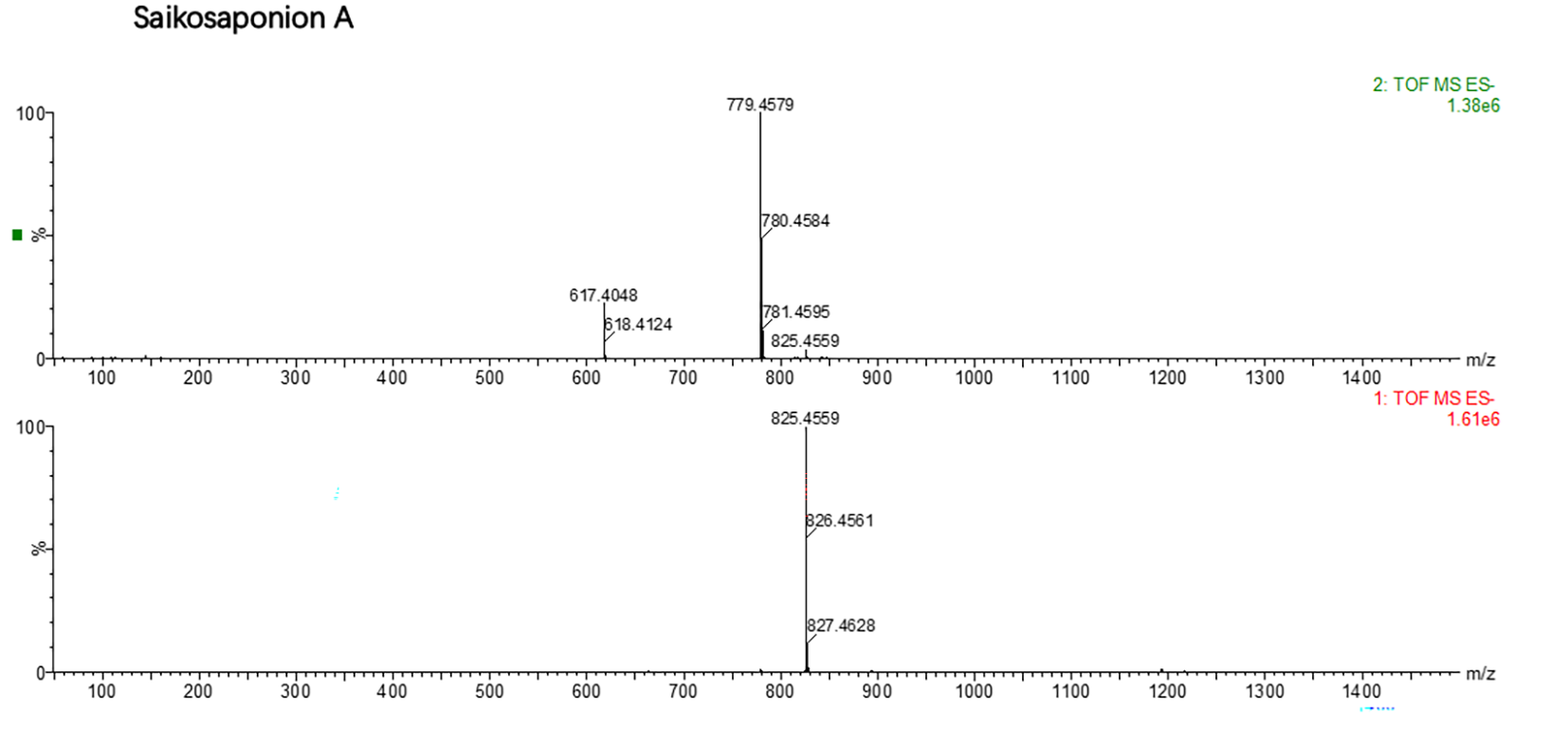

Supplement: Supplementary file 4 [file Image5.PNG]

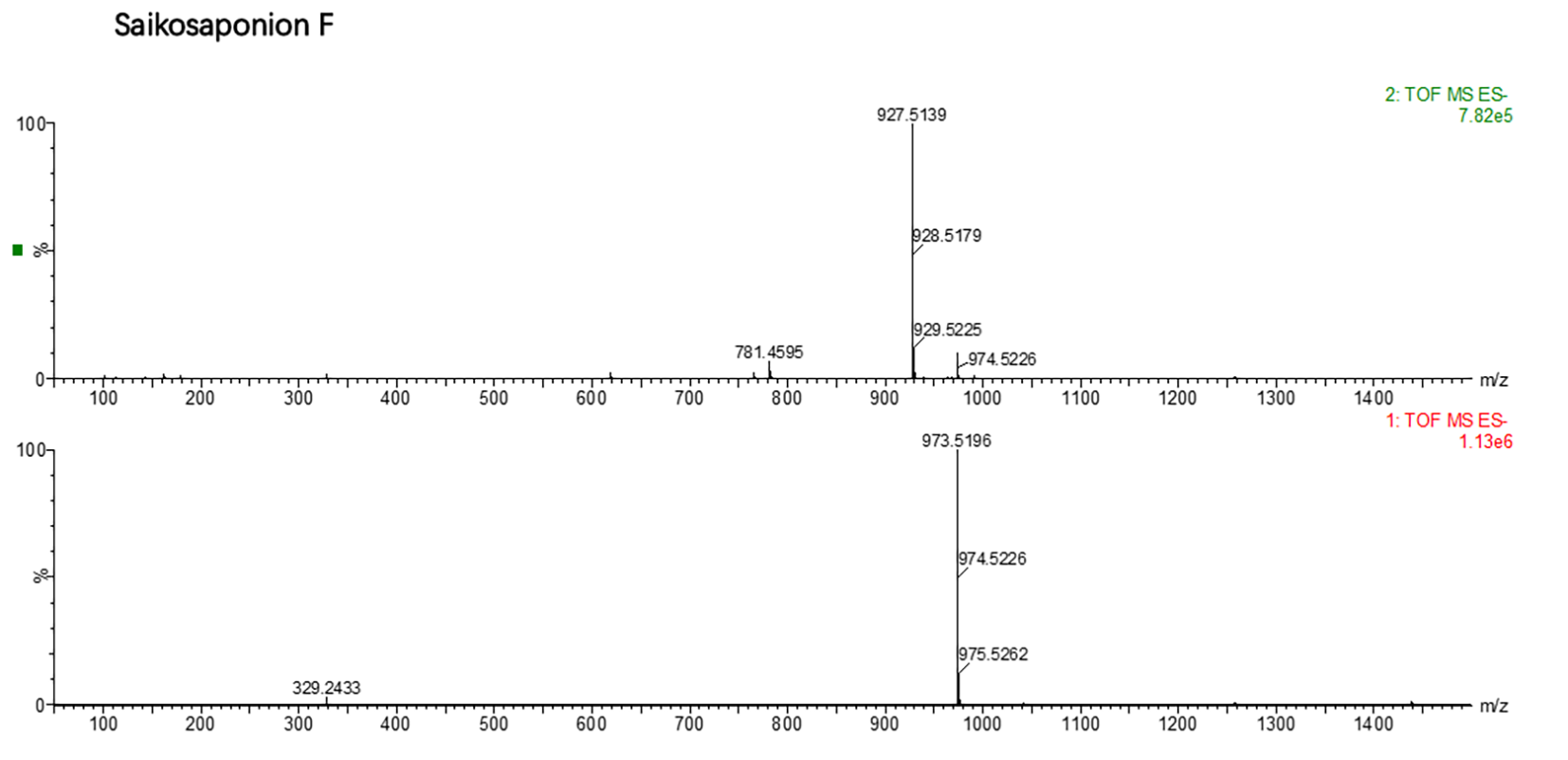

Supplement: Supplementary file 5 [file Image4.PNG]

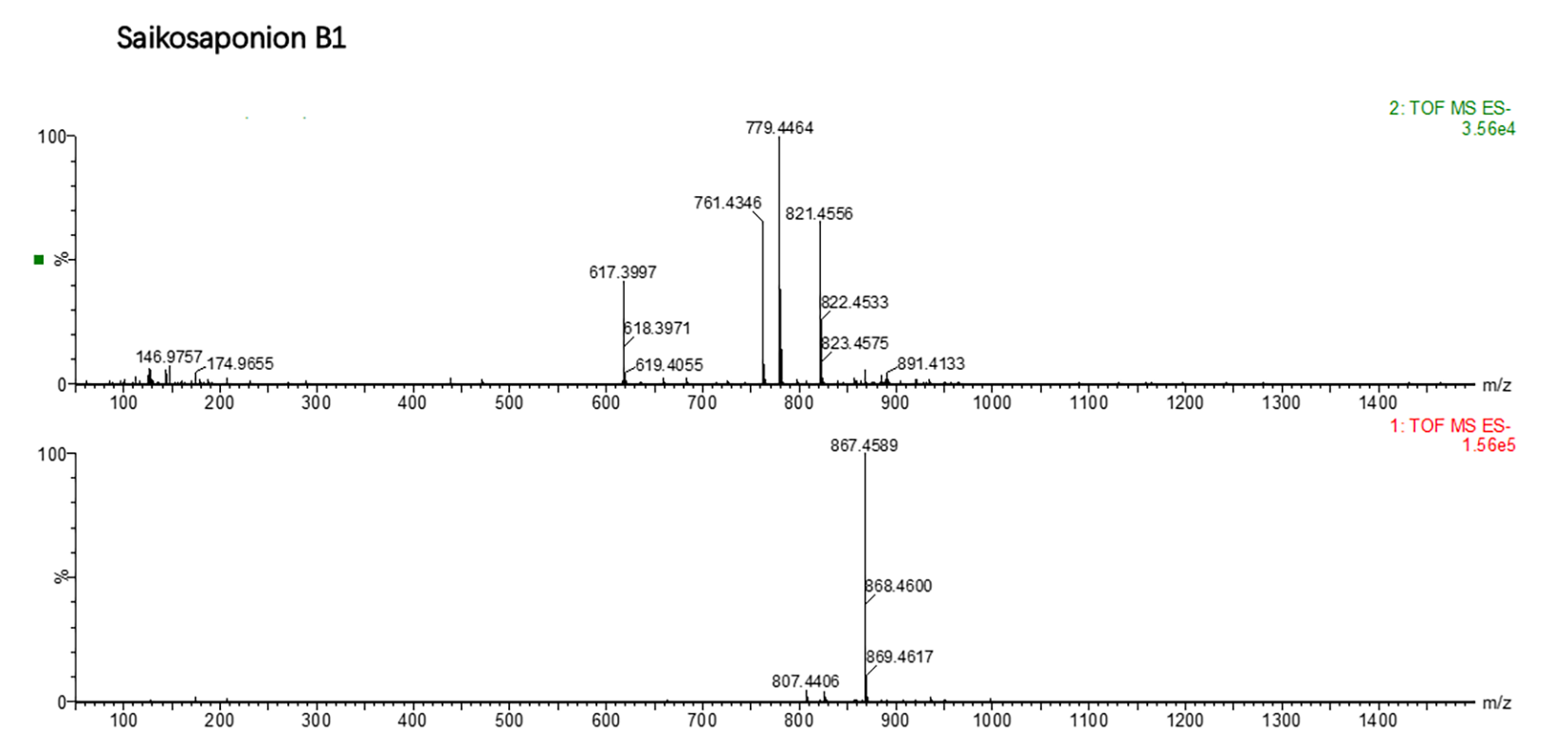

Supplement: Supplementary file 6 [file Image7.PNG]

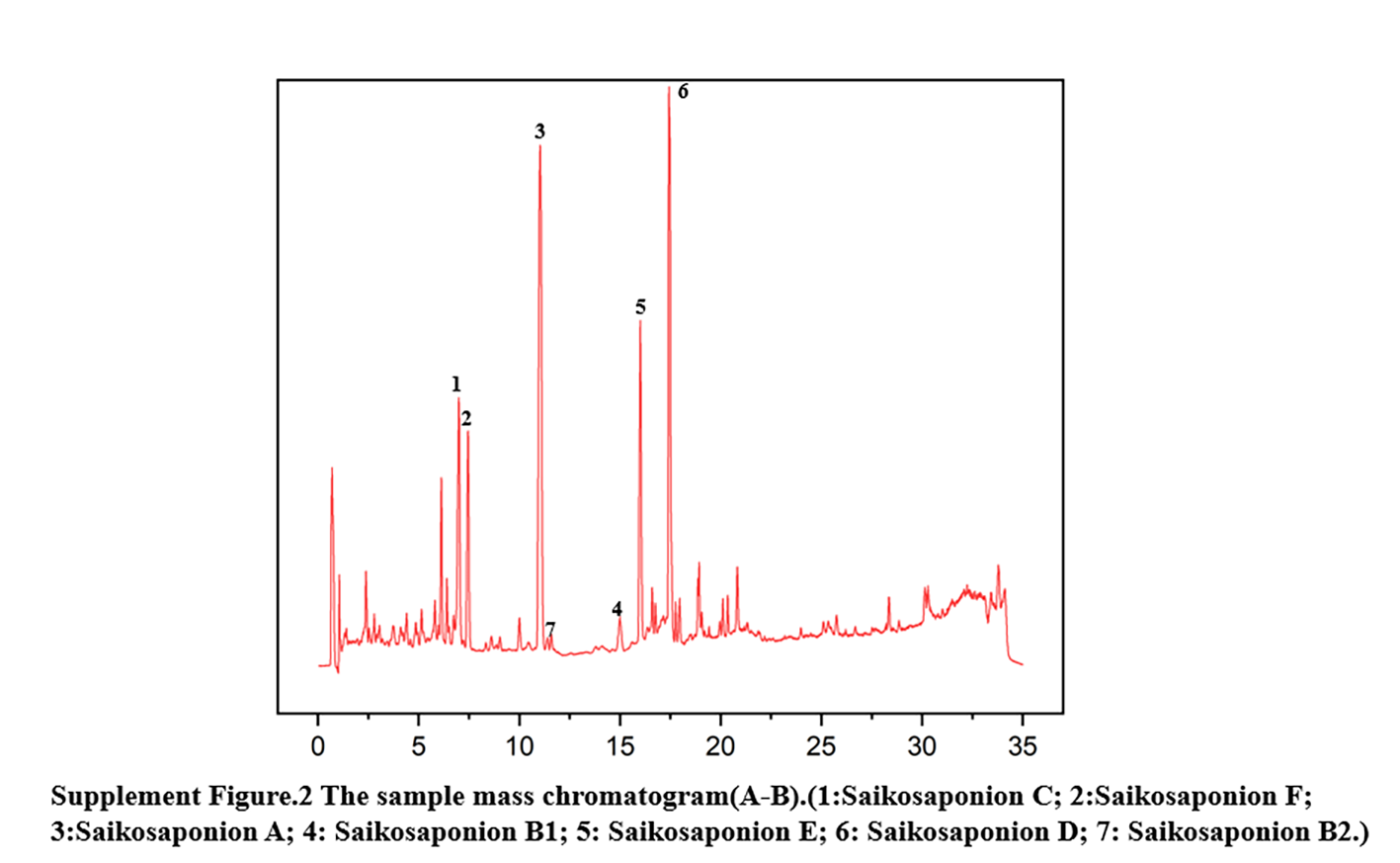

Supplement: Supplementary file 7 [file Image2.PNG]

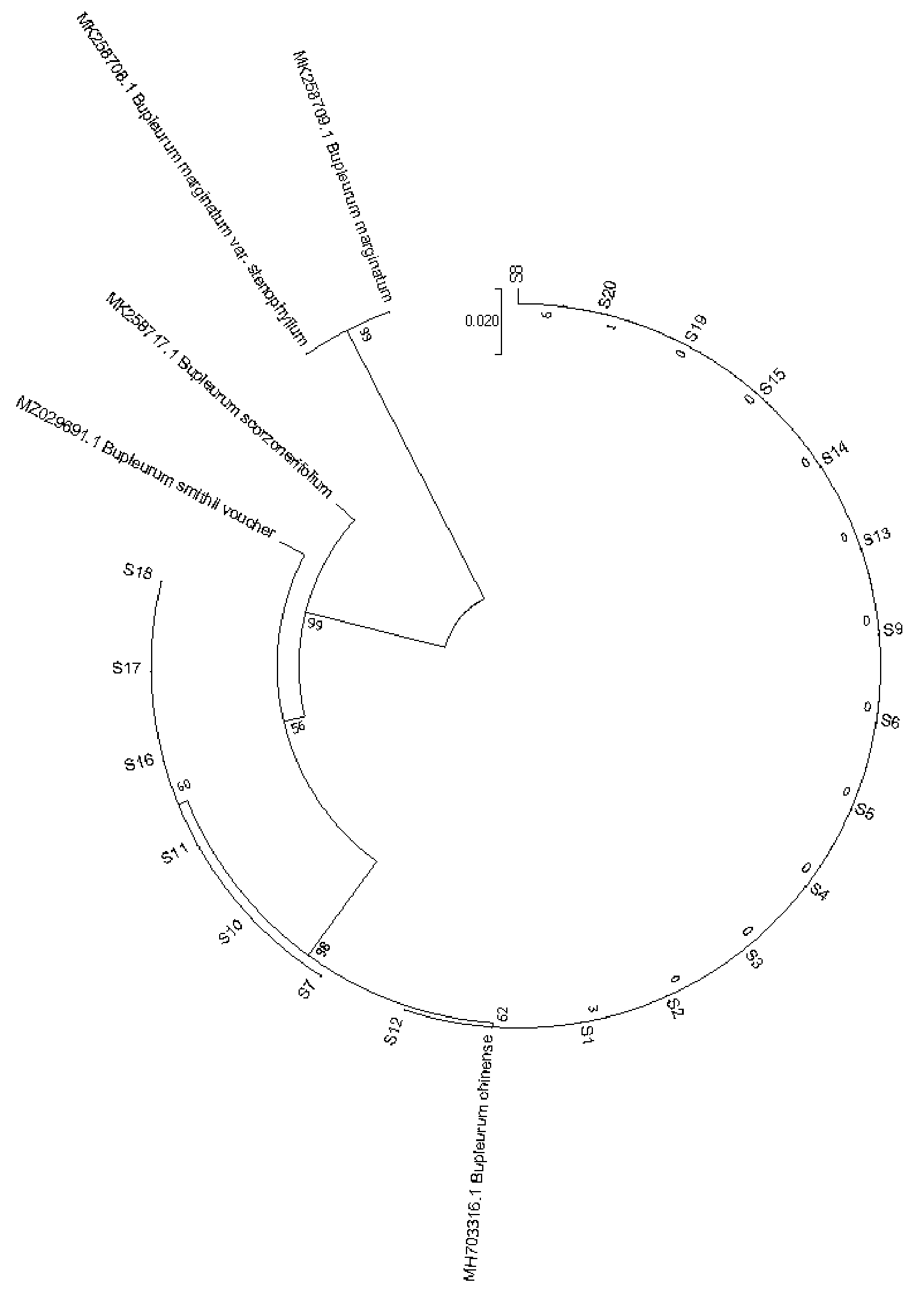

Supplement: Supplementary file 9 [file Image1.PNG]

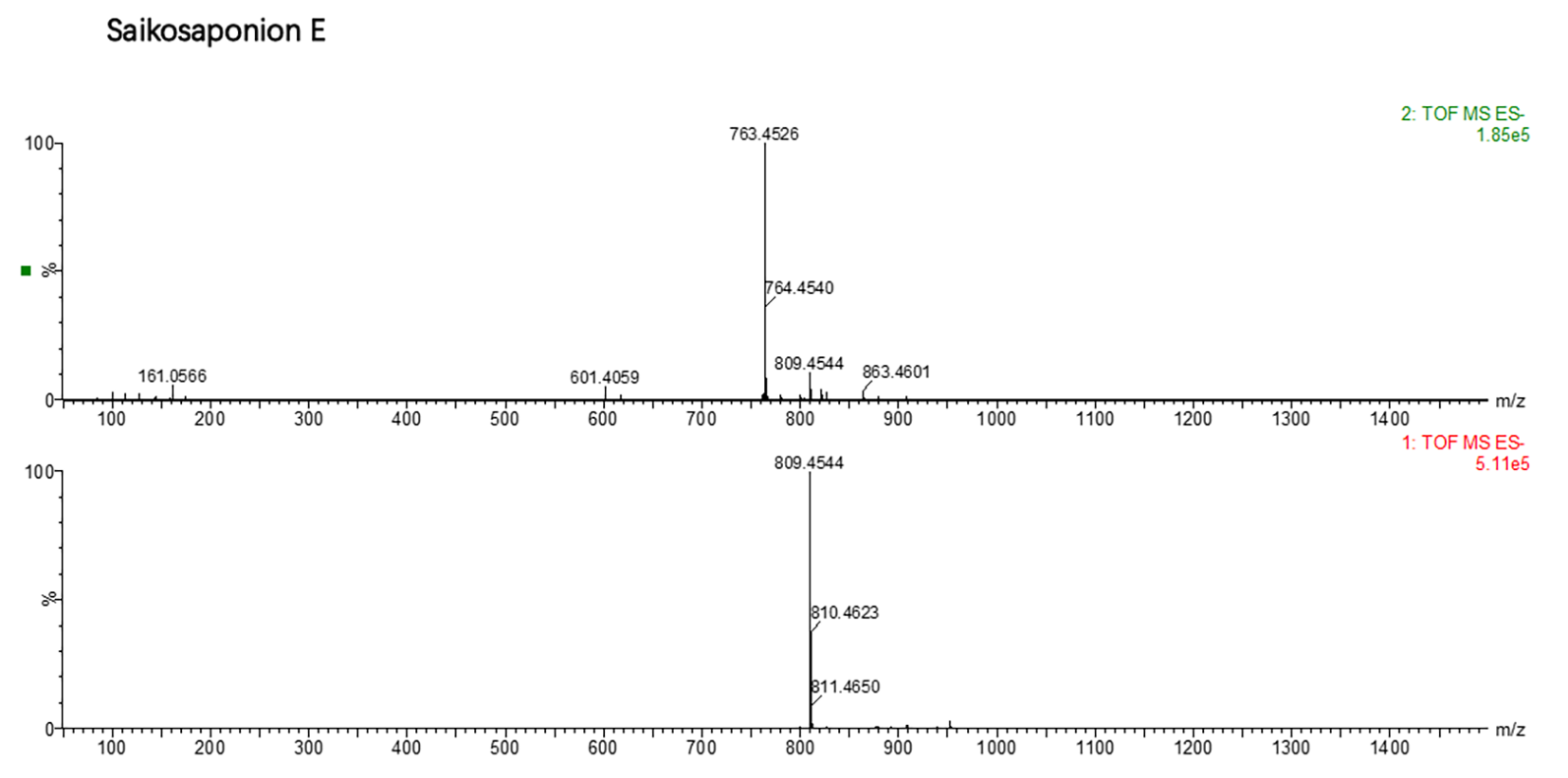

Supplement: Supplementary file 10 [file Image8.PNG]

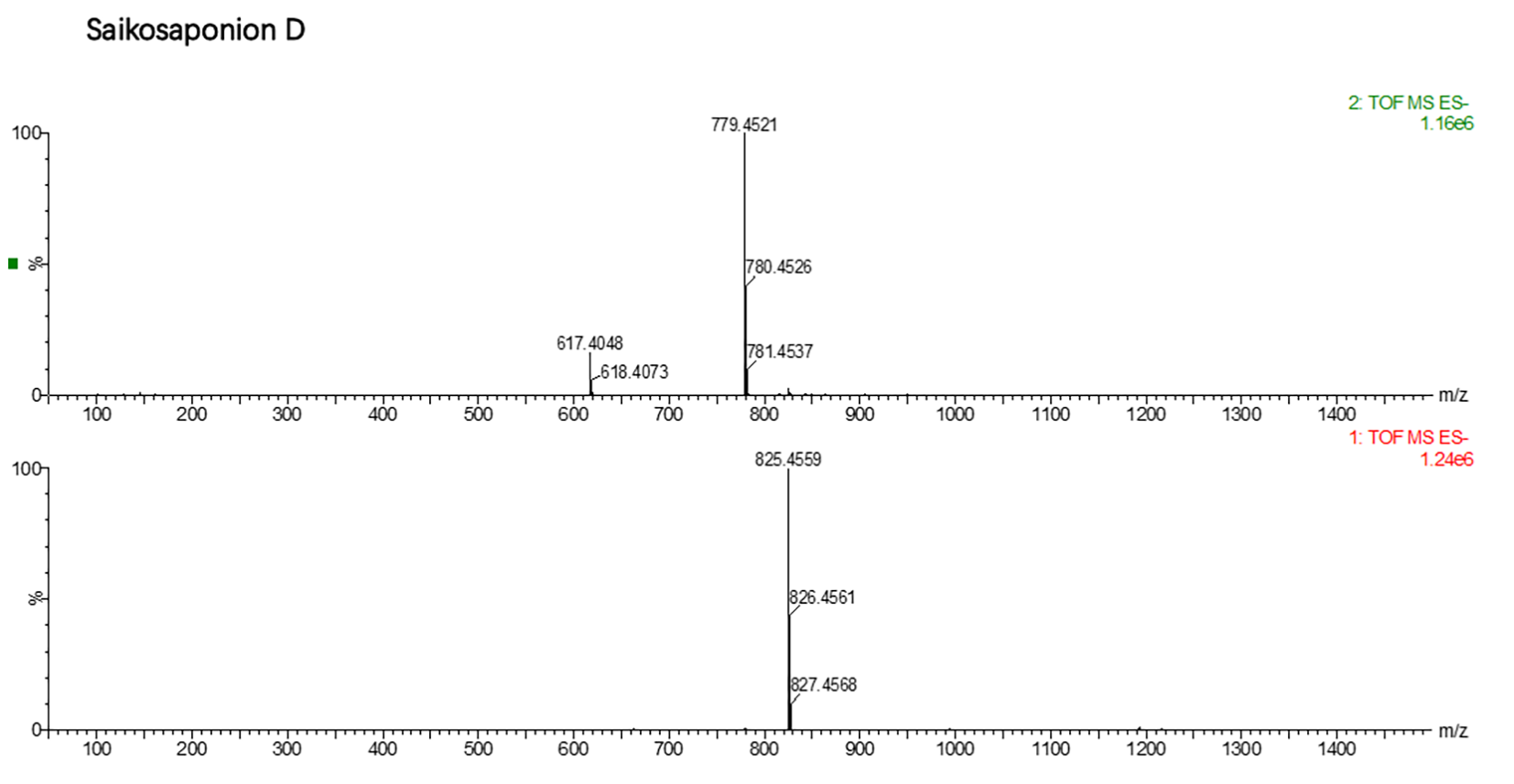

Supplement: Supplementary file 11 [file Image9.PNG]

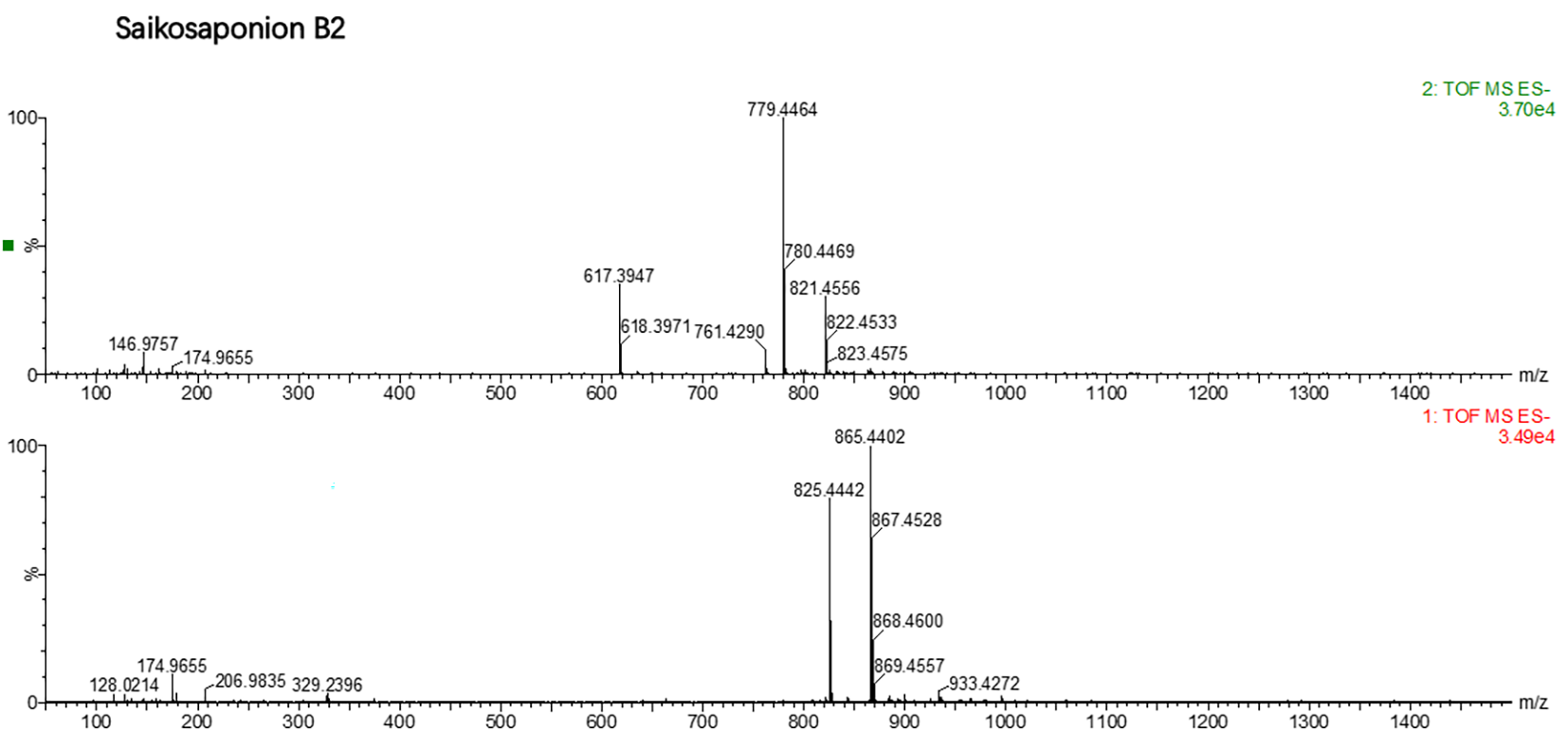

Supplement: Supplementary file 12 [file Image6.PNG]

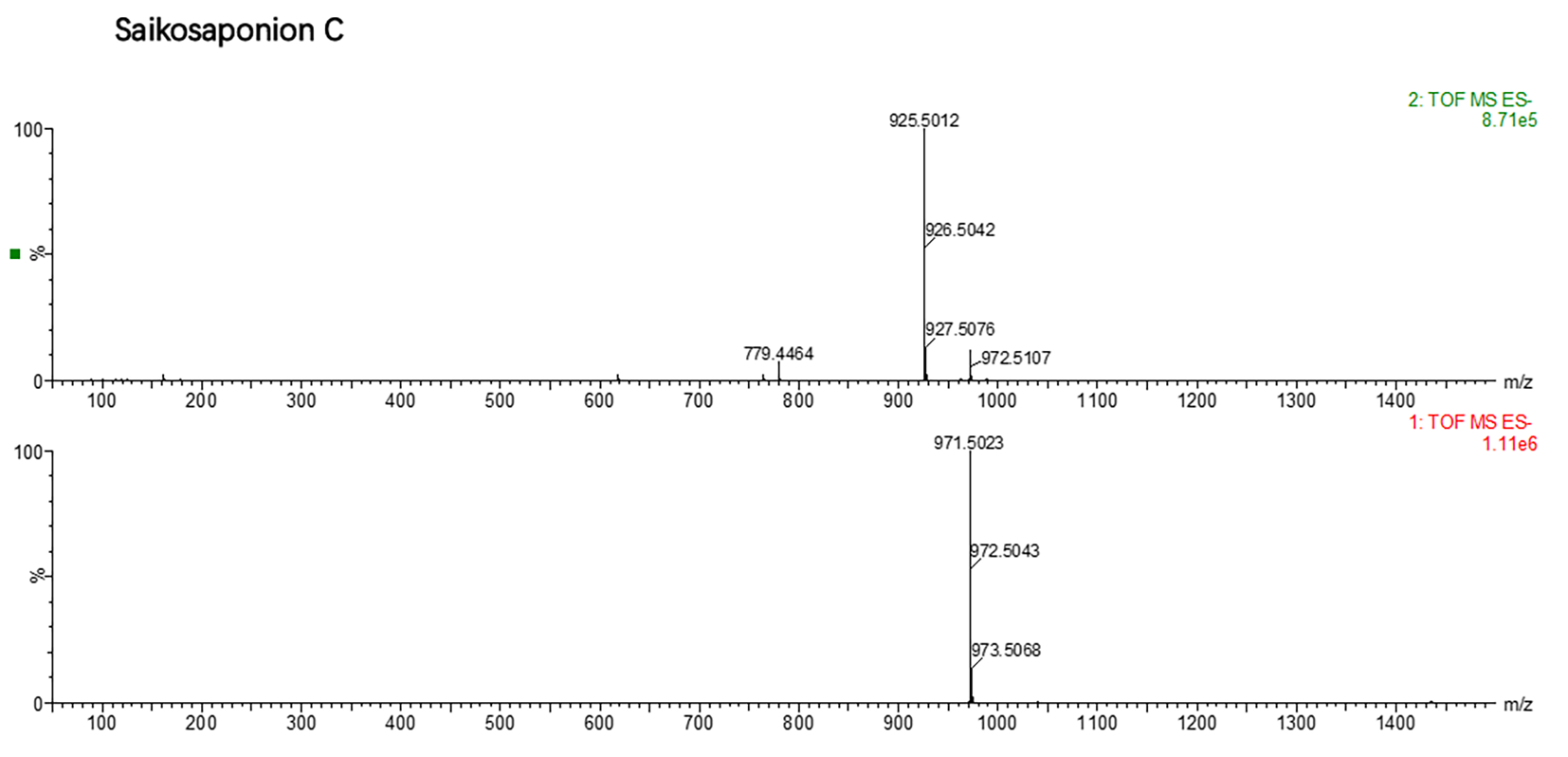

Supplement: Supplementary file 13 [file Image3.PNG]
